# Cross-Sectional Associations between Prenatal Per- and Poly-Fluoroalkyl Substances and Bioactive Lipids in Three Environmental Influences on Child Health Outcomes (ECHO) Cohorts

**DOI:** 10.1021/acs.est.4c00094

**Published:** 2024-05-01

**Authors:** Himal Suthar, Tomás Manea, Dominic Pak, Megan Woodbury, Stephanie M. Eick, Amber Cathey, Deborah J. Watkins, Rita S. Strakovsky, Brad A. Ryva, Subramaniam Pennathur, Lixia Zeng, David Weller, June-Soo Park, Sabrina Smith, Erin DeMicco, Amy Padula, Rebecca C. Fry, Bhramar Mukherjee, Andrea Aguiar, Sarah Dee Geiger, Shukhan Ng, Gredia Huerta-Montanez, Carmen Vélez-Vega, Zaira Rosario, Jose F. Cordero, Emily Zimmerman, Tracey J. Woodruff, Rachel Morello-Frosch, Susan L. Schantz, John D. Meeker, Akram N. Alshawabkeh, Max T. Aung, on behalf of Program Collaborators for Environmental Influences on Child Health Outcomes

**Affiliations:** † Department of Population and Public Health Sciences, University of Southern California, Los Angeles, California 90032, United States; ‡ Department of Civil and Environmental Engineering, Northeastern University, Boston, Massachusetts 02115, United States; § Gangarosa Department of Environmental Health, Emory University Rollins School of Public Health, Atlanta, Georgia 30322, United States; ∥ Department of Environmental Health Sciences, University of Michigan School of Public Health, Ann Arbor, Michigan 48109, United States; ⊥ Institute for Integrative Toxicology, Michigan State University, East Lansing, Michigan 48824, United States; # Department of Food Sciences and Human Nutrition, Michigan State University, East Lansing, Michigan 48824, United States; ∇ Department of Pharmacology and Toxicology, Michigan State University, East Lansing, Michigan 48824, United States; ○ College of Osteopathic Medicine, Michigan State University, East Lansing, Michigan 48824, United States; ◆ Department of Internal Medicine-Nephrology, University of Michigan, Ann Arbor, Michigan 48824, United States; ¶ Department of Molecular and Integrative Physiology, University of Michigan, Ann Arbor, Michigan 48109, United States; †† NSF International, Ann Arbor, Michigan 48105, United States; ‡‡ Environmental Chemistry Laboratory, Department of Toxic Substances Control, California Environmental Protection Agency, Berkeley, California 94710, United States; §§ Program on Reproductive Health and the Environment, University of California, San Francisco, San Francisco, California 94143, United States; ∥∥ Department of Environmental Sciences and Engineering, University of North Carolina, Chapel Hill, Gillings School of Global Public Health, Chapel Hill, North Carolina 27599, United States; ⊥⊥ Department of Biostatistics, University of Michigan School of Public Health, Ann Arbor, Michigan 48109, United States; ## Beckman Institute for Advanced Science and Technology, University of Illinois Urbana−Champaign, Champaign, Illinois 61801, United States; ∇∇ Department of Comparative Biosciences, University of Illinois Urbana−Champaign, Champaign, Illinois 61802, United States; ○○ Department of Kinesiology and Community Health, University of Illinois at Urbana−Champaign, Champaign, Illinois 61801, United States; ◆◆ Department of Epidemiology and Biostatistics, University of Georgia, Athens, Georgia 30606, United States; ¶¶ University of Puerto Rico Graduate School of Public Health, San Juan, Puerto Rico 00935, United States; ††† Department of Communication Sciences and Disorders, Northeastern University, Boston, Massachusetts 02115, United States; ‡‡‡ Department of Environmental Science, Policy and Management and School of Public Health, University of California, Berkeley, Berkeley, California 94720, United States

**Keywords:** PFAS, mixtures, bioactive lipids, eicosanoids, pregnancy outcomes, inflammatory pathways, metabolic pathways

## Abstract

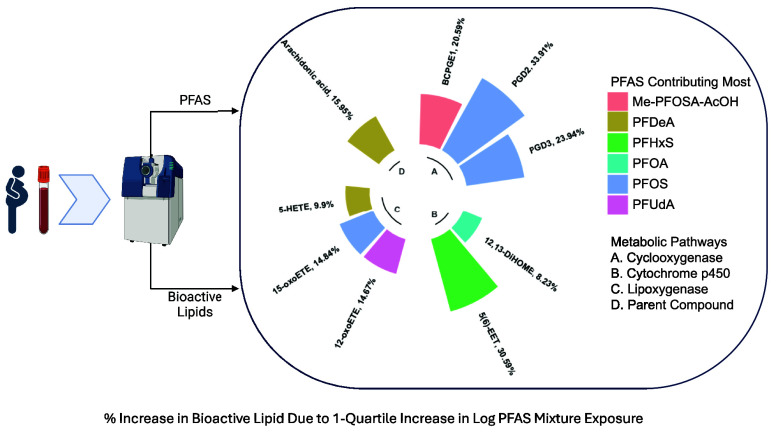

Prenatal per- and poly-fluoroalkyl substances (PFAS) exposure may influence gestational outcomes through bioactive lipids—metabolic and inflammation pathway indicators. We estimated associations between prenatal PFAS exposure and bioactive lipids, measuring 12 serum PFAS and 50 plasma bioactive lipids in 414 pregnant women (median 17.4 weeks’ gestation) from three Environmental influences on Child Health Outcomes Program cohorts. Pairwise association estimates across cohorts were obtained through linear mixed models and meta-analysis, adjusting the former for false discovery rates. Associations between the PFAS mixture and bioactive lipids were estimated using quantile g-computation. Pairwise analyses revealed bioactive lipid levels associated with PFDeA, PFNA, PFOA, and PFUdA (*p* < 0.05) across three enzymatic pathways (cyclooxygenase, cytochrome p450, lipoxygenase) in at least one combined cohort analysis, and PFOA and PFUdA (*q* < 0.2) in one linear mixed model. The strongest signature revealed doubling in PFOA corresponding with PGD2 (cyclooxygenase pathway; +24.3%, 95% CI: 7.3–43.9%) in the combined cohort. Mixture analysis revealed nine positive associations across all pathways with the PFAS mixture, the strongest signature indicating a quartile increase in the PFAS mixture associated with PGD2 (+34%, 95% CI: 8–66%), primarily driven by PFOS. Bioactive lipids emerged as prenatal PFAS exposure biomarkers, deepening insights into PFAS’ influence on pregnancy outcomes.

## Abstract 2

Exposure to per- and poly-fluoroalkyl substances (PFAS), ubiquitous in consumer and industrial products, was revealed to be associated with elevated levels of bioactive lipids across three enzymatic pathways in pregnant women.

## 1 Introduction

Widespread environmental contamination of per- and poly-fluoroalkyl substances (PFAS) poses a major public health concern. Human exposure to PFAS can occur through ingestion of contaminated food and water, and inhalation of indoor air contaminated by consumer products.^1^ Biomonitoring in the National Health and Nutrition Examination Study (NHANES) has reported over 98% detection of PFAS compounds in serum of U.S. residents.^2^ PFAS metabolism and excretion is slow due to their carbon–fluorine bonds, with *in vivo* half-lives spanning from several months to years.^3^ Increasing experimental and epidemiological evidence indicates adverse health effects attributable to PFAS exposure, including kidney dysfunction, hormone disruption, and liver, reproductive, and developmental toxicity.^4^ Biomonitoring of PFAS in NHANES and the Environmental influences on Child Health Outcomes (ECHO) Program indicates moderate correlation between individual compounds, highlighting the need to consider PFAS as a mixture to evaluate cumulative health effects and ameliorate residual confounding.^5,6^

PFAS exposures during pregnancy, a sensitive period of the life course, have been identified as potential risk factors for adverse birth outcomes and pregnancy complications. One systematic review of prenatal PFAS exposures identified select compounds associated with increased odds of preterm birth and miscarriage.^7^ There is also evidence indicating disparities in associations between PFAS exposure in women and chronic health outcomes, with one study revealing that Black women (median age 49) had higher risk of developing hypertension compared to White women.^8^ Our team also identified increased depressive symptoms associated with higher PFAS exposure among immigrant pregnant women compared to U.S. born counterparts (median age 34) in the Chemicals in Our Bodies cohort.^9^ Widespread evidence of human exposure, coupled with associated health effects, warrants detailed investigation into the intermediate mechanisms of PFAS toxicity to inform risk assessment and develop potential interventions.

Bioactive lipids, including polyunsaturated fatty acids such as arachidonic acid, are metabolized by conserved families of enzymes (e.g., cytochrome p450s, lipoxygenases, and cyclooxygenases) yielding secondary eicosanoid metabolites with important downstream physiological functions.^10^ Eicosanoids partly regulate inflammation and influence cardiovascular and renal function, and perturbations in circulating eicosanoids are potential biomarkers of adverse pregnancy outcomes.^11−13^ In a metabolomic study in Atlanta, Georgia, lipid metabolism pathways were associated with gestational age at birth, including linoleic acid, a bioactive lipid compound.^14^ We have also shown in a previous LIFECODES cohort study that higher concentrations of several eicosanoid metabolites derived from the cytochrome p450, lipoxygenase, and cyclooxygenase enzymes were associated with increased risk of spontaneous preterm birth.^15^ Another study in the LIFECODES cohort identified higher concentrations of eicosanoid metabolites from the cytochrome p450 and lipoxygenase pathways associated with greater risk of being born small for gestational age.^16^

PFAS have been found to be associated with changes to several biological pathways, including metabolism of bioactive lipids, amino acids, and xenobiotic detoxification.^17^ This is corroborated by experimental studies indicating that PFAS interfere with cytochrome p450 signaling, critical for cellular metabolism.^18,19^ PFAS also interfere with homeostasis of intracellular calcium gradients, impacting calcium-dependent enzymatic activity and altering systemic bioactive lipid profiles.^20^ Evidence that PFAS are linked to whole pathways of lipid metabolism provides an impetus for deeper investigation of targeted lipid metabolites associated with PFAS, particularly during pregnancy.

The objective of this study was to quantify maternal PFAS exposures and bioactive lipids during pregnancy and estimate the individual and cumulative associations of PFAS with these biomarkers. There is a need for modern epidemiology studies to integrate greater racial, socioeconomic, and geographic diversity to better inform risk estimation across historically marginalized communities. Thus, our current study utilized a diverse sample across three birth cohorts in the ECHO Program. Our study hypothesized that higher concentrations of PFAS are associated with increased eicosanoid metabolite concentrations based on previous studies indicating that higher concentrations of these biomarkers are associated with adverse birth outcomes.

## 2 Methods

### 2.1 Study Populations

The ECHO Program integrates diverse sample populations into a large cohort to advance research of how environmental factors from preconception through childhood influence child health and development.^21^ To support this program goal, this study integrates data across three ECHO samples (Table 1): Chemicals in Our Bodies (CiOB), Illinois Kids Development Study (IKIDS), and the ECHO-PROTECT cohort.^22,23^ These three sample populations were selected due to having rich data on PFAS, targeted lipid biomarkers, and neurodevelopment measures as part of the Opportunities and Infrastructure Fund. The samples utilize demographic diversity across the U.S. and serve as a foundation to expand measurements in future studies across additional ECHO samples. This approach has been applied in previous studies within the ECHO program with robust sensitivity analyses to account for different cohort characteristics.^24,25^

**Table 1:** Demographic Profile of CiOB, IKIDS, and ECHO-PROTECT Cohorts

			cohort			
characteristic		overall	CiOB, *N* = 73^a^	IKIDS, *N* = 287^a^	ECHO-PROTECT, *N* = 54^a^	*p*-value^b^
mother’s Race						<0.001
	non-Hispanic white	253 (62%)	34 (47%)	219 (78%)	0	
	black	19 (4.6%)	-	14 (5.0%)	0	
	Asian	48 (11.7%)	14 (19%)	34 (12%)	0	
	Hispanic	78 (19%)	17 (23%)	7 (2.5%)	54 (100%)	
	other	11 (2.7%)	-	8 (2.8%)	0	
	missing	5	0	5	0	
maternal age (years)		31.8 (28.8, 34.5)	33.1 (29.9, 36.0)	31.8 (29.1, 34.3)	28.0 (24.0, 33.0)	<0.001
	missing	6	1	0	5	
maternal education						<0.001
	<HS	11 (2.7%)	-	-	-	
	HS/GED/some college	77 (19%)	16 (22%)	39 (14%)	22 (45%)	
	Bachelors	142 (35%)	15 (21%)	111 (39%)	16 (33%)	
	Graduate	179 (44%)	37 (51%)	135 (47%)	7 (14%)	
	missing	5	0	0	5	
prepregnancy BMI (kg/m^2^)		25 (22, 29)	24 (22, 27)	25 (22, 30)	25 (21, 29)	0.2
	missing	20	11	1	8	
parity						<0.001
	0	191 (47%)	35 (51%)	156 (54%)	0 (0%)	
	1 or more	214 (53%)	34 (49%)	131 (46%)	49 (100%)	
	missing	9	4	0	5	
maternal household Income						<0.001
	<$50,000	110 (28%)	18 (26%)	52 (18%)	40 (93%)	
	$50,000–$99,999	145 (36%)	6 (9%)	137 (48%)	-	
	>$100,000	143 (36%)	45 (65%)	97 (34%)	-	
	missing	16	4	1	11	
gestational age at visit (weeks)		17.4 (16.6, 18.9)	23.1 (19.7, 25.7)	17.0 (16.4, 17.7)	25.7 (24.4, 27.9)	<0.001
	missing	10	0	0	10	

a *n* (%), cells with counts ≤5 have been masked; median (IQR).

b Kruskal–Wallis rank sum test; Pearson’s Chi-squared test

The CiOB cohort participants are based in San Francisco, CA, and were recruited during the second trimester of pregnancy from three University of California San Francisco hospitals. Women included in the CiOB cohort had to be ≥18 years of age, primarily speak English or Spanish, and have a singleton pregnancy. The IKIDS cohort participants were recruited between 10 and 14 weeks gestation from two obstetric clinics in Champaign-Urbana, Illinois. Women included in the IKIDS cohort had to be between 18 and 40 years of age, have English fluency, have a singleton pregnancy, be ≤15 weeks gestation at enrollment, not have a child already in the IKIDS cohort, reside within a 30 min drive from the University of Illinois Urbana-Campaign (UIUC) campus, and plan to remain in the area through the child’s first birthday. The ECHO-PROTECT cohort participants were recruited before 20 weeks’ gestation from two hospitals and five clinics in the Northern Karst aquifer region in Puerto Rico. Enrollment of the cohort began in 2011 and is ongoing. Inclusion criteria for the women in this cohort included being between 18 and 40 years of age, residing in the Northern Karst aquifer region, not having used oral contraceptives three months prepregnancy, not having undergone in vitro fertilization, and having no major pre-existing conditions. Detailed information on study recruitment and data collection per cohort has been previously described.^22,23^ Participants in each cohort provided written informed consent for inclusion in this study, and local institutional review boards per cohort reviewed and approved study protocols. Sample sizes for each cohort are described in greater detail below, and the flow diagram for selection of the final analytical data set used in statistical analyses is illustrated in Supplemental Figure 1.

### 2.2 PFAS Exposure Assessment

In the CiOB (*n* = 73, median 23 weeks’ gestation at sample collection) and IKIDS (*n* = 287, median 17 weeks’ gestation at sample collection) cohorts, 12 PFAS compounds were quantified in maternal serum during pregnancy: perfluorononanoic acid (PFNA), perfluoroheptanoic acid (PFHpA), perfluorodecanoic acid (PFDeA), perfluorododecanoic acid (PFDoA), perfluorooctanoic acid (PFOA), perfluorooctanesulfonamide (PFOSA), 2-(*N*-methyl-perfluorooctane sulfonamido) acetic acid (Me-PFOSA-AcOH), 2-(*N*-ethyl-perfluorooctane sulfonamido) acetic acid (Et-PFOSA-AcOH), perfluoroundecanoic acid (PFUdA), perfluorohexanesulfonate (PFHxS), perfluorooctanesulfonic acid (PFOS), and perfluorobutanesulfonic acid (PFBS). After sample collection, serum was frozen at −80 °C. Samples were processed at the Environmental Chemical Laboratory at the California Department of Toxic Substances Control using a previously described analytical protocol.^26^ PFAS quantification was achieved using internal standards per serum sample and an automated online solid phase extraction method coupled to liquid chromatography and tandem mass spectrometry. The limits of detection for individual PFAS compounds were equal to three times the standard deviation of the blank negative control sample.^26^ In each batch of serum PFAS analyses, analytes were quantified using a constructed calibration, and calibration curve regression coefficients (*R*^2^) of 0.98 to 0.99 were obtained. We utilized in-house quality control materials that were prepared by spiking a known amount of PFAS compounds in blank bovine serum at low and high levels, 4 samples each, achieving over 90% recovery and coefficient of variation (CV%) values ranging from 5.2 to 14.1. There was also a QC/QA effort to evaluate the interlaboratory comparison. Standard reference materials (SRM 1958) were utilized from the National Institute of Standards and Technology (Gaithersburg, MD), and quality control samples spiked with known PFAS concentrations were from the United States Centers for Disease Control and Prevention as reference materials. Blank samples of bovine serum (Hyclone/GE Healthcare LifeSciences) were also processed for each sample batch, and no PFAS were detected above their respective limit of detection in these blank samples.

Maternal serum samples from the ECHO-PROTECT cohort (*n* = 54, median 26 weeks’ gestation at sample collection) were used to quantify nine PFAS compounds (Table 1). After sample collection, serum was frozen at −80 °C and processed at NSF International (Ann Arbor, MI). PFAS quantification was also performed by liquid chromatography and tandem mass spectrometry, simulating the Centers for Disease Control and Prevention’s poly-fluoroalkyl chemicals laboratory procedure method No: 6304.1. Standards of known purity and identity were used during preparation of calibration, quality control, and internal standards, with a minimum of 5 standards for each compound of interest. The validated analyte calibration curve correlation coefficient (*R*^2^) ranges were ≥0.996. The method accuracy (% nominal concentration) and precision (% relative standard deviation [RSD]) were determined through six replicate analyses of analytes spiked at three different concentrations in pooled human serum across validation runs on three separate days (*n* = 18), reflecting the intraday and interday variability of the assay. Calibration checks were conducted after every 10 samples. The accuracy (% nominal concentration) range across all analytes was 95.1–103% with the precision (%RSD) range for the serum quality control samples across all analytes being 2.3–16%.

Method detection limits for all PFAS measured in each cohort are reported in Supplemental Table 1. For samples with values below the limit of detection, we used machine-read values (if reported) or concentrations imputed using the limit of detection divided by the square root of 2.^27^ Of the PFAS compounds measured, we selected to analyze those with a detection rate of ≥70%, highlighted in Supplemental Table 1 by cohort.

### 2.3 Bioactive Lipids Assay

Maternal plasma samples were used to quantify a targeted panel of 50 bioactive lipids (Supplemental Table 2) in the CiOB (*n* = 73, median 24 weeks’ gestation at sample collection), IKIDS (*n* = 287, median 17 weeks’ gestation at sample collection), and PROTECT (*n* = 54, median 26 weeks’ gestation at sample collection) cohorts. For ECHO-PROTECT and CiOB cohorts, plasma was collected using ethylenediaminetetraacetic acid plasma tubes and temporarily stored at +4 °C for less than 4 h. Blood was subsequently centrifuged for 20 min and stored at −80 °C. Women in the IKIDS cohort provided a fasted (10 to 12 h) blood sample collected in green-top sodium heparin tubes, which were kept at room temperature for 2 h prior to processing and centrifuged at room temperature for 20 min. The resulting plasma was aliquoted immediately into cryovials for storage at −80 °C. The targeted bioactive lipids panel consisted of five parent fatty acid compounds and 45 eicosanoid metabolites derived from three enzymatic groups (lipoxygenases, cytochrome p450s, and cyclooxygenases), and full names and abbreviations are documented in Supplemental Table 2. Quantification of bioactive lipid concentrations was achieved using a 6490 Triple Quadrupole mass spectrometer (Agilent, New Castle, DE), where the mass spectrometer was set to a targeted multiple reaction monitoring mode and individual biomarkers were identified based on metabolite-specific fragmentation and retention time. Limits of detection were not calculated across individual instrument analysis cycles, and all measured bioactive lipids had machine-read values. Further information on instrumental parameters and quality control/assurance have been previously documented.^28^ Briefly, we conducted sequential dilution of each internal standard in duplicate to establish linearity and estimate the coefficient of variation (CV) of the measurements at various concentrations. CV values for a majority (86%) of bioactive lipids were <40% indicating modest levels of variability, and hence measurements are of high confidence and likely highly reproducible. Six bioactive lipids (13,14-D-PGJ2, PGD3, PGE3, BCPGE2, BCPGE1, 9-oxoODE) were at the threshold of limit of detection potentially accounting for the higher CV/variability observed (65 to 112%); higher sensitivity methods may enhance reproducibility of these analytes. We ran a pool of study samples at the beginning of each batch and after each 12 samples during mass spectrometry to assess drift in measurements over time and the batch-to-batch variability.

### 2.4 Statistical Analyses

We utilized a conceptual model and directed an acyclic graph of the relationships between PFAS and bioactive lipids (Supplemental Figure 2) to inform our statistical analyses. We tabulated distributions of key covariates from our conceptual model for each cohort in our study. Spearman correlations were estimated between all high-detect (i.e., ≥70% of observations exceeding minimum level of detection in cohort) PFAS compounds and bioactive lipids within each cohort. Combined cohort correlations were not conducted due to differences in high-detect PFAS across cohorts. Based on which PFAS were highly detected in each cohort (Supplemental Table 1), associations were estimated in combined cohort samples with two (CiOB and IKIDS) or three cohorts (CiOB, IKIDS, and ECHO-PROTECT) using linear mixed effect regressions (random intercept for cohort) and meta-analyses. Significance was evaluated at a level of α ≤ 0.05. Associations between PFAS mixtures and bioactive lipids were estimated using quantile g-computation in the combined IKIDS and CiOB cohort.^29^ Analyses are described in detail below and were performed with *R* (version 4.3.0).

#### 2.4.1 Combined Cohort Analysis

Linear mixed effects models were utilized to test combined cohort pairwise associations between all sampled bioactive lipids and common high-detection PFAS, using natural log-transformed lipid levels as outcomes and natural log-transformed PFAS levels as exposures. These linear mixed effects models included a random intercept for the cohort to partly ameliorate bias from different laboratory analyses of PFAS, heterogeneity in demographic characteristics between cohorts, and differences in PFAS and bioactive lipid concentrations between cohorts. All effect estimates were transformed to enhance interpretability and represent a percent change in a given bioactive lipid corresponding to a doubling (or 100% increase) of individual PFAS (exact transformation executed: (2^β^ – 1)*100). Unadjusted and adjusted random intercept models were generated for all three cohorts by using PFAS common to all three cohorts: PFNA and PFOS (Supplemental Table 1), and when combining the CiOB and IKIDS cohorts, using the natural log-transformed high-detect PFAS common to those two cohorts as exposures (Supplemental Table 1). Adjusted models included variables selected *a priori* based on our previous investigation of PFAS and biomarkers of lipid peroxidation and oxidative stress.^30^ The covariates included in the adjusted models were maternal age, education, prepregnancy BMI, parity, and gestational age at sampling of bioactive lipids. Maternal education was specifically included due to its establishment as a proxy for social and economic resources,^31^ and its associations with both PFAS concentrations during pregnancy^32^ and metabolic perturbations.^33^ To evaluate the appropriateness of utilizing *a priori* confounders, we estimated associations between the covariates and PFAS and bioactive lipids (Supplemental Tables 4–6). In the CiOB and IKIDS cohorts, all covariates had a significant association with at least one PFAS and one bioactive lipid using a *p*-value threshold of 0.10; in the ECHO-PROTECT cohort, maternal age and maternal education were significantly associated with at least one PFAS and one bioactive lipid, using a *p*-value threshold of 0.15 due to limited sample size and power in this cohort. Missing observations for the covariates were omitted from the adjusted models. *Q*-values were calculated for all pairwise models to control for false discovery rates,^34^ and, to test for nonlinear associations between PFAS and bioactive lipid levels, we conducted a sensitivity analysis treating quartiles of log-transformed PFAS levels as exposures in our linear mixed effect models.

#### 2.4.2 Meta-Analysis

Each cohort in our study has heterogeneous demographic features and varying exposure levels of PFAS. Thus, to recognize cohort-specific exposure response functions, we estimated pairwise effect estimates for PFAS and bioactive lipids from each individual cohort using linear regression, adjusted for the same covariates modeled in the combined cohort analysis except for ECHO-PROTECT, which excluded parity due to 0% variance within the cohort. We combined these effect estimates by meta-analysis using the METAL method,^35^ yielding overall effect estimates and *p* values for pairwise associations between individual bioactive lipids and common high-detect PFAS. Meta-analysis was implemented for all three cohort-specific models, and for the IKIDS and CiOB cohort-specific models. Interpretation of cohort-specific effect estimates was treated as a sensitivity analysis to evaluate consistencies in combined cohort and meta-analysis and reported in the Supporting Information.

#### 2.4.3 PFAS Mixtures Analysis

To address the study goal of estimating the cumulative effect of multiple PFAS on bioactive lipids, we used quantile g-computation,^29^ a generalization of the weighted quantile sum (WQS) regression method^36,37^ which does not assume directional homogeneity of the exposures’ effects on the outcome. This method outputs an unbiased estimate of the effect on a particular bioactive lipid associated with simultaneously increasing all PFAS exposures by one quartile. Quantile g-computation was implemented in the combined CiOB and IKIDS sample to measure the mixtures effect of log-transformed high-detect PFAS common to both the CiOB and IKIDS cohorts (Et-PFOSA-AcOH, PFDEA, PFHxS, PFNA, PFOA, PFOS, and PFUDA**;**Supplemental Table 1) on all log-transformed bioactive lipids, controlling for the same covariates included in the adjusted combined CiOB and IKIDS random intercept model. The cohort itself was included as an additional fixed effect covariate to account for any between-cohort differences.^30^ To assess the robustness of our main results with respect to our selected covariates, we conducted a vibration analysis of the primary effect estimate, obtaining β coefficients when including all possible combinations of four out of the five of our covariates in our main quantile g-computation model. To assess the effect of missingness in covariates (Table 1) on our main effect estimate, we conducted another vibration analysis, performing multiple imputation on the CiOB and IKIDS combined sample using 10 iterations and comparing the outputted β coefficients to that of our main quantile g-computation model. The resulting β coefficients from both vibration analyses were evaluated based on significance and whether they were in the 95% confidence interval of our main results.

## 3 Results

### 3.1 Descriptive Statistics

Table 1 shows the distribution of demographics and gestational age at plasma collection for bioactive lipids across all three cohorts. White women represented the largest group in CiOB (47%) and IKIDS (78%), and ECHO-PROTECT had only Hispanic women (100%). Women in CiOB were the oldest (median 33 years), followed by IKIDS (median 32 years), and ECHO-PROTECT (median 28 years; *p* < 0.01). Women in IKIDS had the highest educational attainment, with 86% having completed a bachelor’s or graduate degree, followed by CiOB (72%), and ECHO-PROTECT (47%; *p* < 0.01). All mothers in ECHO-PROTECT reported having at least one previous birth—higher than CiOB (49%) and IKIDS (46%; *p* < 0.01). The annual household income for women in CiOB was the highest with 65% reporting annual household incomes of ≥$100,000, then women in IKIDS (34%), while 93% of women in ECHO-PROTECT reported a household income <$50,000 (*p* < 0.01). Income was not adjusted for local cost of living or median salary. Prepregnancy BMI was similar across all three cohorts (median 24 kg/m^2^ in CIOB and 25 kg/m^2^ in IKIDS and ECHO-PROTECT, *p* = 0.2).

Distributions of PFAS in our sample and in the 2011–2018 NHANES cycle sampling adult women of reproductive age and bioactive lipids by cohort are reported in Supplemental Tables 1 and 3, respectively. Among the high-detect PFAS common to all three cohorts, PFNA (median 0.20 ng/mL) and PFOS (median 1.78 ng/mL) concentrations were the lowest in the ECHO-PROTECT cohort (*p* < 0.01), while the CiOB cohort had the highest median concentration of PFNA (0.53 ng/mL), and the IKIDS cohort had the highest median concentration of PFOS (3.27 ng/mL). Among the high-detect PFAS common to only the CiOB and IKIDS cohorts, the greatest differences were observed for PFUdA (median 0.18 ng/mL in CiOB and median 0.06 ng/mL in IKIDS; *p* < 0.01) and PFHxS (median 0.55 ng/mL in CiOB and median 0.76 ng/mL in IKIDS; *p* = 0.02). There were differences in the interquartile range of PFAS levels in our sample and those in the NHANES sample for PFNA, PFHpA, PFHxS, and PFBS. Among the bioactive lipids, we observed the greatest difference across cohorts for linoleic acid concentrations (median 668 μMol/L in CiOB, 1185 μMol/L in IKIDS, and 102 μMol/L in ECHO-PROTECT; *p* < 0.01) and 20-carboxy arachidonic acid (CAA) concentrations (median 185 nMol/L in CiOB, 302 nMol/L in IKIDS, and 74 nMol/L in ECHO-PROTECT; *p* < 0.01).

### 3.2 Within-Cohort Correlations

Within-cohort Spearman correlations between bioactive lipids and PFAS are shown in Supplemental Figure 3A for the CiOB cohort, in Supplemental Figure 3B for the IKIDS cohort, and in Supplemental Figure 3C for the ECHO-PROTECT cohort. In the CiOB cohort, correlation coefficients between bioactive lipids and PFAS ranged between −0.31 and 0.38, with the strongest negative correlation between DHA and PFOS, and the strongest positive correlation between 13S-HODE and PFUDA. For bioactive lipids in the CiOB cohort, correlation coefficients ranged from −0.47 (16-HETE and 13-oxoODE) to 1 (12(13)-EpoME and 13S-HODE), while correlation coefficients for PFAS were between −0.08 (PFDeA and Et-PFOSA-AcOH) and 0.81 (PFNA and PFOA). In the IKIDS cohort, correlation coefficients between bioactive lipids and PFAS ranged between −0.14 and 0.22, with the strongest negative correlation between 15-HETE and PFHxS, and the strongest positive correlation between arachidonic acid and PFNA. Correlation coefficients for bioactive lipids in the IKIDS cohort were between −0.75 (13,14-D-PGJ2 and 18-HETE) and 0.89 (9S-HODE and 13S-HODE), and between 0.05 (PFOA and Me-PFOSA-AcOH) and 0.75 (PFNA and PFOA) for PFAS. In the ECHO-PROTECT cohort, correlation coefficients between bioactive lipids and PFAS ranged between −0.37 and 0.32, with the strongest negative correlation between 9,10-DiHOME and PFNA, and the strongest positive correlation between 13,14-D-PGJ2 and PFNA. Bioactive lipid correlation coefficients within the ECHO-PROTECT cohort were between −0.59 (PGA2 and 11-HETE) and 0.8 (9(10)-EpoME and 12(13)-EpoME), and the correlation coefficients between PFNA and PFOS in the ECHO-PROTECT cohort was 0.57.

### 3.3 Pairwise Associations between Individual PFAS and Bioactive Lipids

**Figure 1:**
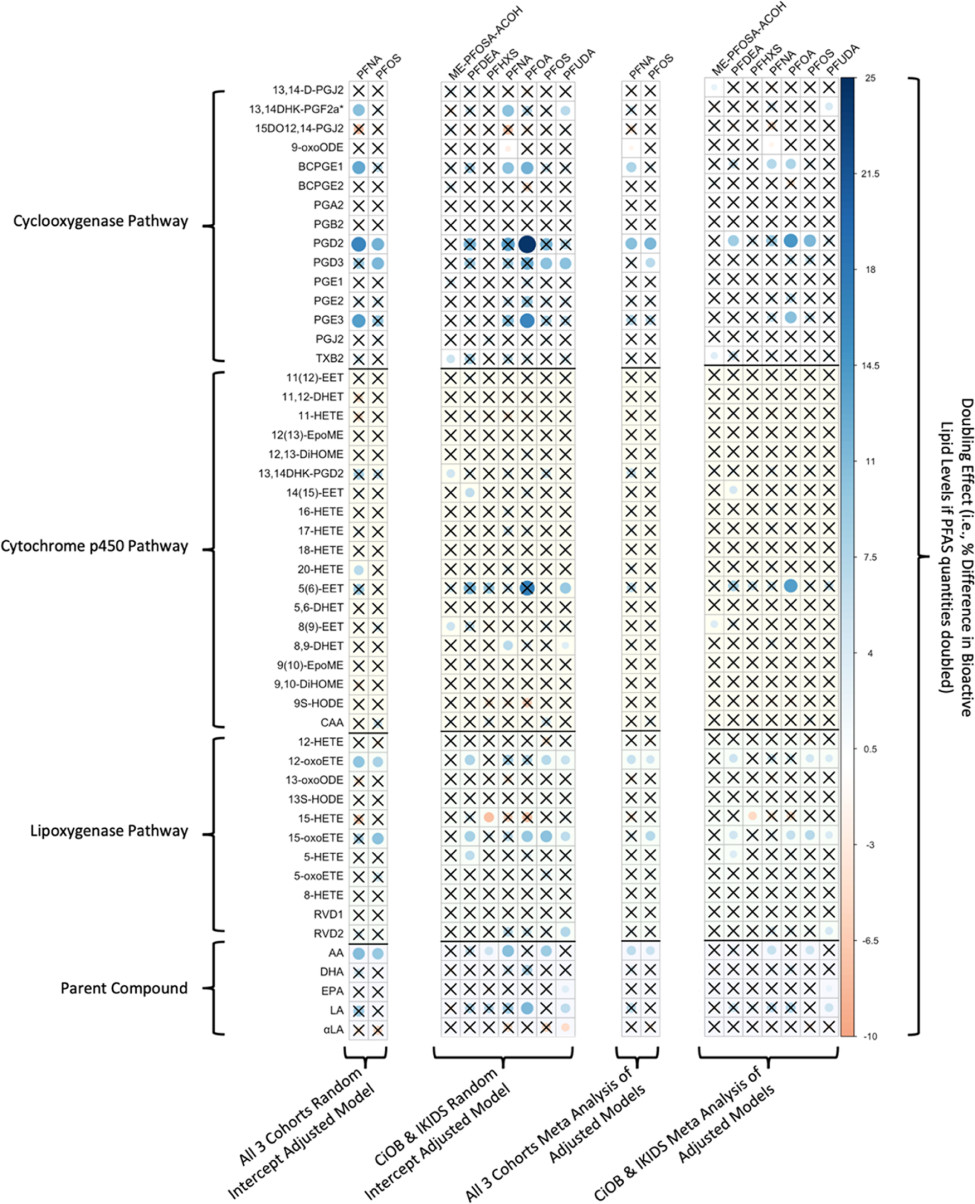
Heatmap of β estimates corresponding to percentage change in bioactive lipids as a result of doubling log-transformed PFAS for adjusted joint analyses and meta-analyses run on adjusted within-cohort models. The magnitude of effect estimates in each cell in the heatmap corresponds to the intensity of the color band in the legend. Nonsignificant values (*p* > .05) are marked with black “X”. Sample sizes range from 343 to 383 (see Supplemental Tables 7, 8, 9, and 10 for exact pairwise sample sizes).

The majority of significant associations observed in at least one of the four combined models were positive. All coefficients representing the pairwise associations between bioactive lipids and PFAS from the adjusted random intercept models and meta-analyses are presented in a heatmap (Figure 1) as the effect per doubling of PFAS concentration. Additional output of these models, including exact *p* values and standard errors can be found in Supplemental Tables 7–10.

In the cyclooxygenase pathway, 15 significant associations between bioactive lipids and PFAS were observed in at least one of the four combined cohort models. The association between BCPGE1 and PFNA was significant in all four models (doubling effect in PFNA range of 7.2–12.8% increase in BCPGE1 across models). The associations between 9-oxoODE and PFNA (doubling effect in PFNA range 1.6–2.4% decrease in 9-oxoODE), PGD2 and PFOS (doubling effect in PFOS range 11.3–11.9% increase in PGD2), and PGD3 and PFOS (doubling effect in PFOS range 6.8–11.5% increase in PGD3) were significant in three models.

In the cytochrome p450 pathway, no significant associations were found common to all four models; however, there were significant positive associations between 14(15)-EET and PFDeA (doubling effect range 4.6–6.4%) and between 8(9)-EET and Me-PFOSA-AcOH (doubling effect range 4.0–5.4%) observed in the CiOB and IKIDS random intercept model and in the meta-analysis integrating effect estimates from CiOB and IKIDS cohorts.

Within the lipoxygenase pathway, significant positive associations were observed between 12-oxoETE and PFOS (doubling effect range 4.9–8.2%) and 15-oxETE and PFOS (doubling effect range 7.0–10.4%) across all four models.

Among the parent compounds, significant positive associations were observed for arachidonic acid and PFNA (doubling effect range 6.0–10.9%) and arachidonic acid and PFOS (doubling effect range 5.5–9.7%) across all four models.

We explored multiple testing comparison adjustments in the linear mixed effects models (Supplemental Tables 7 and 8). While none were below a threshold of 0.1 in the two-cohort or three-cohort models, there were seven PFAS and bioactive lipid pairs (PGD2 and PFOA; PGD3 and PFUdA; 13,14DHK-PGF2a* and PFUdA; 12-oxoETE and PFUdA; 15-oxoETE and PFUdA; 15-oxoETE and PFOS; arachidonic acid and PFOS) below the 0.2 threshold in the two-cohort linear mixed effect models. Our sensitivity analysis of within-cohort pairwise associations can be found in Supplemental Tables 11–16. While individual cohort analyses are underpowered to detect associations, directions of pairwise associations between individual cohorts and combined cohorts were largely consistent. Quartile analyses of the 3-cohort linear mixed effect models revealed that while certain quartiles of log-transformed PFAS may drive the overall direction of association, there is no substantial evidence of nonlinear associations (Supplemental Table 17). Quartile analyses of the two-cohort linear mixed effect models are suggestive of nonlinear relationships between PFOA and PGD2 and 13,14-PGJ2 (Supplemental Table 18).

### 3.4 PFAS Mixture Associations

**Figure 2:**
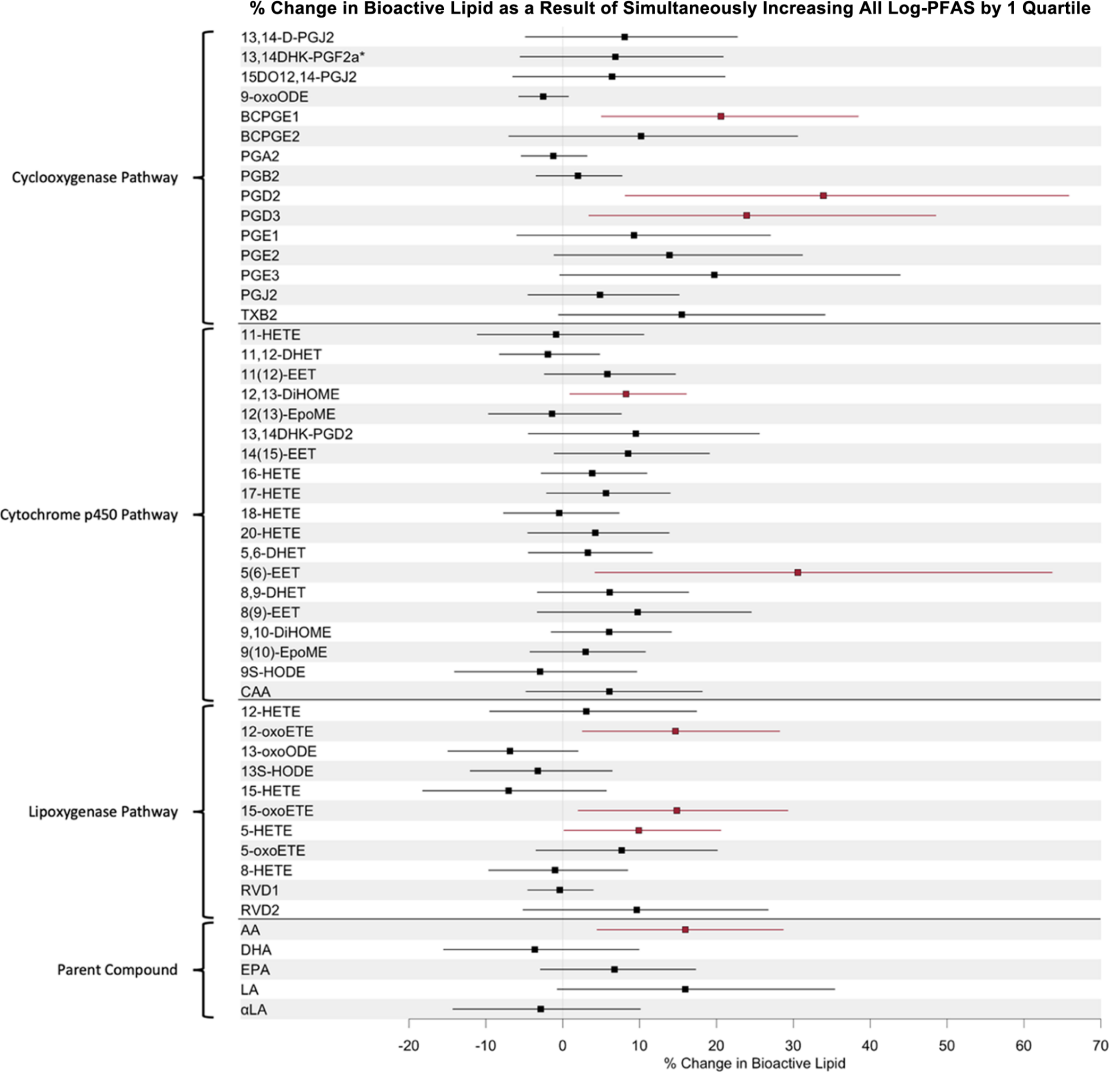
Forest plot of quantile g-computation effect estimates in the combined cohort analysis with IKIDS and CIOB cohorts (*N* = 343), showing of β estimates corresponding to percentage change in bioactive lipids associated with a simultaneous 1-quartile increase in all log-transformed PFAS. Model adjusted for maternal age, maternal education, prepregnancy BMI, parity, gestational age at visit, and cohort.

Quantile g-computation utilizing the CiOB and IKIDS cohorts indicated that simultaneously increasing all log-transformed PFAS in the mixture (PFNA, PFDeA, PFoA, Me-PFOSA, PFUdA, PFHxS, and PFOS) by one quartile corresponded to increases in BCPGE1, PGD2, and PGD3 (cyclooxygenase pathway); 12,13-DiHOME and 5(6)-EET (cytochrome p450 pathway); 12-oxoETE, 15-oxoETE, and 5-HETE (lipoxygenase pathway); and the arachidonic acid parent compound. The largest increase in the cyclooxygenase pathway was observed in PGD2 (34% increase, 95% CI [8%, 66%]), with decomposition of the effect estimate indicating that PFOS exhibited the greatest positive weight to the overall mixture effect among other PFAS compounds (Supplemental Table 19). In the cytochrome p450 pathway, the largest increase associated with a simultaneous one quartile increase in all PFAS in the mixture was in 5(6)-EET (31% increase, 95% CI [4%, 64%]), with PFHxS contributing most to this increase. The largest increase in the lipoxygenase pathway was observed in 15-oxoETE (15% increase, 95% CI [2%, 29%]), with PFOS having the largest contribution. In the parent compounds, arachidonic acid had a 16% increase (95% CI [4%, 29%]), driven predominantly by PFOS. No significant decreases in bioactive lipids were observed. These effects are visualized in Figure 2 and reported in detail in Supplemental Table 19. Our vibration analyses assessing the robustness of our covariate selection and degree to which our models were affected by missingness in covariate data did not yield any effect estimates that were outside of the 95% CI of our main model (Supplemental Tables 20–21).

## 4 Discussion

### 4.1 Summary of Findings across Statistical Approaches

In the present study, we estimated associations between PFAS and bioactive lipids in single pollutant and mixture models, utilizing cohort stratified analyses, combined cohort analyses, and meta-analyses. Combined cohort analyses and meta-analyses identified mostly positive associations of PFAS with parent fatty acid compounds and their secondary eicosanoid metabolites derived from the lipoxygenase, cytochrome p450, and cyclooxygenase pathways. Mixtures analysis using quantile g-computation revealed that the PFAS mixture exhibited positive associations with bioactive lipids across all three enzymatic pathways, and with the exception of 12(13)-DiHOME, at least one individual PFAS was associated with these bioactive lipids in the single pollutant models for either combined cohort analyses or meta-analyses. Largely consistent results between mixtures analysis and pairwise associations strengthen confidence in targeted bioactive lipids as potential mechanistic biomarkers of PFAS exposure, providing insight into addressing the health effects of PFAS as an entire class. Although our estimated associations are susceptible to false-positive associations, our findings allow for the prioritization of pairs of PFAS and bioactive lipids for future hypothesis testing and replication in independent samples.

### 4.2 Biological Context of Associations in Bioactive Lipid Enzymatic Pathways

The physiological implications of our findings vary by the metabolic pathway investigated. We observed the strongest positive effect between the PFAS mixture and cyclooxygenase-derived PGD2, which was consistent with individual PFAS compound analyses for PFDeA, PFOA, and PFOS in the CiOB and IKIDS meta-analyses. Systemic inflammation and oxidative stress are both antecedent physiological states conferring risk of adverse pregnancy outcomes such as spontaneous preterm birth and preeclampsia.^38,39^ Cyclooxygenases have been implicated in promoting inflammation through the production of prostaglandins^40^ and prostaglandin production is sensitive to reactive oxygen species and oxidative stress imbalances.^41^ Existing literature indicates that cyclooxygenases are important for regulating reproductive health and fetal development; animal models have found that deficiencies in the genes encoding these enzymes cause altered implantation, increased mortality, and impaired organ development in offspring.^42^ Animal studies indicate that disruptions in cyclooxygenase function and prostaglandin synthesis can also lead to altered neurodevelopment and behavior^43,44^—a previous study in an experimental rat model found that the PGD2 signaling pathway is involved in neuroinflammation, and induction of this pathway results in neurodegenerative pathologies.^45^ Evidence from experimental mechanistic studies, combined with our findings that PFAS are linked to altered eicosanoid concentrations within the cyclooxygenase pathway, highlights this pathway as a potential mediator between PFAS and adverse pregnancy outcomes.

We observed that the PFAS mixture was associated with two cytochrome p450-derived eicosanoids: 12,13-DiHOME and 5(6)-EET. Cytochrome p450 enzymes have varied regulatory roles, including biosynthesis of endogenous hormones, detoxification of xenobiotics, and cellular metabolism.^19^*In vitro* experiments indicate that multiple PFAS compounds can directly interfere and inhibit cytochrome p450 activity.^18^ 12,13-DiHOME has been classified as an oxylipin derived from linoleic acid, and is involved with inflammation, endocrine signaling, and adipogenesis.^46,47^ In a previous case-control study by our team on the LIFECODES cohort using the same bioactive lipids panel measured (median 26 weeks’ gestation) in this present study, we reported that 12,13-DiHOME was associated with increased risk of spontaneous preterm birth (*n*_cases_ = 31, *n*_controls_ = 115).^15^ Further, placental 5(6)-EET has been detected at higher levels in preeclamptic women compared to controls, linking 5(6)-EET to regulation pathways associated with preeclampsia.^48,49^ Additionally, a study of 146 adult women reported that single nucleotide polymorphisms of cytochrome p450 genes amplified cancer risk attributable to PFAS exposures.^50^ While bioactive lipids were not measured in that study, this is a consistent biological inference based on their findings of increased breast cancer risk in association with higher PFOS and PFOA and polymorphisms in cytochrome p450 genes. Based on these previous studies, PFAS potentially interferes directly with cytochrome p450 activity and subsequent eicosanoid metabolite concentrations, and there may be gene and environment interactions. To disentangle these different contributions of PFAS’ effects on lipid metabolism, future studies can consider integrating PFAS, eicosanoids, genotypes, and maternal health outcomes.

Lipoxygenases are calcium-dependent enzymes that catalyze the formation of hydroperoxides from polyunsaturated fatty acids and have been associated with several adverse health outcomes including asthma, skin disorders, and cancers.^51^ We observed that the PFAS mixture was associated with higher levels of the lipoxygenase-derived eicosanoids 12-oxoETE, 15-oxoETE, and 5-HETE, which may be biomarkers for metabolic and cardiovascular disorders. 12-oxoETE has been linked to diabetic macular edema^52^ and experimental models indicate that 15-oxoETE may influence atherosclerosis.^12^ Additionally, our previous study in the LIFECODES cohort identified higher levels of 12-oxoETE and 5-HETE associated with spontaneous preterm birth.^15^ Our present study findings showing that increases in 12-oxoETE and 15-oxoETE were associated with increased PFAS exposure further align with previous experimental evidence showing that PFAS can interfere with intracellular calcium gradients, influencing the catalytic activity of lipoxygenases.^20^ Therefore, future studies should continue to investigate this pathway as a mechanistic link between PFAS exposure and adverse maternal and child health outcomes.

We observed differences in bioactive lipid compound distributions between individual cohorts, possibly driven by variation in genetic makeup of the populations caused by heterogeneous ethnic composition of the cohorts,^53^ or differences in environmental exposures of the cohorts due to diet.^54^ Among the parent polyunsaturated fatty acids, the PFAS mixture was associated with increased concentrations of arachidonic acid. These findings align with the positive signatures that we observed for the secondary eicosanoid metabolites described above derived from these parent compounds. Our reported findings can be partially contextualized with previous metabolomics studies linking PFAS-induced effects to lipid metabolism. One metabolomics study investigated a mixture of six common PFAS (PFOS, PFHxS, PFHpS, PFOA, PFNA, and PFDA) in children and adolescents (*n* = 137) based in Los Angeles, CA, and observed positive associations of the mixture with arachidonic acid and linoleic acid.^55^ Another study of 267 maternal-newborn dyads in Atlanta, GA reported that maternal PFAS exposures were associated with newborn metabolomic signatures for bioactive lipid metabolism including leukotrienes, cytochrome p450 pathway, and linoleic acid.^14^ Our findings align with existing evidence of the deleterious effects of PFAS exposure on pregnancy outcomes, as prenatal/perinatal PFAS exposure has been associated with multiple adverse birth outcomes including preterm birth,^7^ miscarriage, and maternal depression.^9^ Thus, our study underscores the importance of applying targeted metabolomics approaches to investigate the role of lipid metabolism as a potential intermediate mechanism of PFAS exposure on maternal and early child health outcomes.

### 4.3 Implications for Public Health Policy and Practice

In a 2022 report, the National Academies of Science, Engineering, and Mathematics systematically reviewed human health literature on PFAS and produced decision making guideline recommendations for PFAS testing and clinical follow-up,^8^ including screening for hypertensive disorders and lipid panel measures for individuals with serum or plasma PFAS concentrations above 2 ng/mL.^56^ Findings from our present study contextualize these clinical care approaches by providing more granular details on specific prenatal lipid metabolite and PFAS exposure associations. While the bioactive lipids measured in our study have not yet been tested as routine biomarkers in clinical care settings, our findings aid in advancing the foundation for future precision health considerations, as more advanced lipid biomarkers become scalable and tested for clinical utility.

### 4.4 Strengths and Limitations

Our study has notable strengths. First is the diversity of our study population, which included individuals from three distinct geographies, exhibiting heterogeneity across demographics, socioeconomic status, and PFAS exposures. Second, we will discuss our statistical approach. We performed combined cohort analysis utilizing two methods (linear mixed effects models and meta-analysis) and a mixtures analysis using quantile g-computation to assess cumulative associations with PFAS mixtures. The combination of methods applied and the consistency in results across methods strengthens the inference in identified associations. Third, our selection of outcomes was a targeted assay of bioactive lipids not previously tested for associations with PFAS. This targeted assay directly complements existing studies utilizing nontargeted metabolomics by deepening knowledge of specific lipid metabolite features to contextualize larger biological pathways and processes observed in past studies. Combined, this resulted in a robust investigation, with corroboration of our key findings both within our study and with previous literature.

Our study also has limitations to consider. First is the cross-sectional nature of data collection, as serum PFAS and plasma bioactive lipids were measured during the same visit. Single time point assessment is more susceptible to measurement error and reverse causation than longitudinal studies. Because of their long half-lives, PFAS measures may be relatively stable during pregnancy; however, researchers may consider longitudinal designs to more effectively assess spatiotemporal relationships and causal mechanisms driving the observed associations. Additionally, some confounding variables (e.g., maternal education and parity) were heterogeneous across cohorts. There are also potential unmeasured confounders that we did not model in the present study including dietary intake which is known to influence both PFAS and bioactive lipid concentrations; for example, fish and dairy consumption has been linked to increased PFAS levels in humans and is a source of fatty acids including arachidonic acid.^57−59^ Future studies may benefit from considering this confounder via a comprehensive assessment of dietary intake. It is also possible that intermediate underlying metabolic factors such as liver disease or type 2 diabetes are affected by PFAS exposure,^60,61^ which in turn could influence lipid metabolism.^62,63^ Therefore, future studies may consider more complex causal pathways to disentangle the contribution of the underlying maternal metabolic factors in the relationship between PFAS and bioactive lipids. Regarding statistical approaches, we recognize that there are alternatives for analyzing chemical mixtures in health studies. Our study focused on cumulative PFAS mixture associations; however, future studies may consider high-order interactions across multiple exposure variables, latent clustering of correlated exposure variables, or dimension reduction through risk scores based on sources of exposures. We are likely underestimating the effect of PFAS as a whole class, as our study evaluated 12 serum PFAS compounds, and there are thousands of PFAS compounds that humans may be exposed to, especially given the presence of newer replacement and short-chained PFAS compounds.^64^ Future studies should consider nontargeted exposomic approaches to contextualize how associations observed in our study might be influenced by the detection of these PFAS not measured in our study.^65^ Despite this limitation, we highlight the importance of these 12 PFAS compounds being detected at relatively high rates in existing biomonitoring studies—and some are targets for near-term regulation, as the Environmental Protection Agency proposed establishing limits on 6 of the PFAS in our sample (PFOA, PFOS, PFNA, PFHxS, and PFBS) due to their high presence in drinking water in the US.^65^ Limited sample size and detection rates of PFAS in the ECHO-PROTECT cohort reduced our ability to examine differences in associations between Hispanic and White women exposed to PFAS. Finally, we have documented associations between other classes of endocrine-disrupting chemicals (e.g., phthalates, phenols, and parabens) and prenatal bioactive in the LIFECODES cohort (*N* = 173).^66^ It is possible that these chemicals are correlated with prenatal PFAS exposures, contributing to residual confounding and potential interactions and influencing the effects of PFAS on maternal bioactive lipid profiles. Therefore, future investigations should consider broader chemical mixture analyses with bioactive lipids and pregnancy outcomes.

## Data Availability

The R-script used for the present study is found in the Supporting Information. Select deidentified data from the ECHO Program are available through NICHD’s Data and Specimen Hub (DASH). Information on study data not available on DASH, such as some Indigenous data sets, can be found on the ECHO study DASH webpage.

## Supporting Information Available

Flow diagram of final sample selection across CiOB, IKIDS, and PROTECT cohorts; directed acyclic graph of the relationships between maternal per- and poly-fluoroalkyl substances and bioactive lipids; correlation matrices of bioactive lipids and high-detect PFAS in the study cohorts (Appendix A. Supplementary Figures S1–S3) (PDF)
PFAS Detection Rates and Distributions Across Cohorts; Bioactive lipids sampled from maternal plasma; Bioactive Lipid Distribution Across Cohorts; β Estimates and 95% Confidence Intervals for Associations Between Confounders and Bioactive Lipids/PFAS within the CiOB Cohort; β Estimates and 95% Confidence Intervals for Associations Between Confounders and Bioactive Lipids/PFAS within the IKIDS Cohort; β Estimates and 95% Confidence Intervals for Associations Between Confounders and Bioactive Lipids/PFAS within the ECHO-PROTECT Cohort; β Estimates and 95% Confidence Intervals Corresponding to Percentage Change in Bioactive Lipids associated with a Doubling in PFAS for random intercept model across all 3 cohorts, adjusted for Maternal Age, Maternal Education, Pre-Pregnancy BMI, Parity, Gestational Age at Visit; β Estimates and 95% Confidence Intervals Corresponding to Percentage Change in Bioactive Lipids associated (*p* ≤ 0.05) with a Doubling in PFAS for random intercept model across CiOB & IKIDS, adjusted for Maternal Age, Maternal Education, Pre-Pregnancy BMI, Parity, Gestational Age at Visit; β Estimates and 95% Confidence Intervals Corresponding to Percentage Change in Bioactive Lipids associated (*p* ≤ 0.05) with a Doubling in PFAS for meta-analysis across all 3 cohorts, adjusted for Maternal Age, Maternal Education, Pre-Pregnancy BMI, Parity, Gestational Age at Visit; β Estimates and 95% Confidence Intervals Corresponding to Percentage Change in Bioactive Lipids associated (*p* ≤ 0.05) with a Doubling in PFAS for meta-analysis across CiOB & IKIDS, adjusted for Maternal Age, Maternal Education, Pre-Pregnancy BMI, Parity, Gestational Age at Visit; β Estimates and 95% Confidence Intervals Corresponding to Percentage Change in Bioactive Lipids associated with a Doubling in PFAS for CiOB within-cohort models, unadjusted; β Estimates and 95% Confidence Intervals Corresponding to Percentage Change in Bioactive Lipids associated with a Doubling in PFAS for CiOB within-cohort models, adjusted for Maternal Age, Maternal Education, Pre-Pregnancy BMI, Parity, Gestational Age at Visit; β Estimates and 95% Confidence Intervals Corresponding to Percentage Change in Bioactive Lipids associated with a Doubling in PFAS for IKIDS within-cohort models, unadjusted; β Estimates and 95% Confidence Intervals Corresponding to Percentage Change in Bioactive Lipids associated with a Doubling in PFAS for IKIDS within-cohort models, adjusted for Maternal Age, Maternal Education, Pre-Pregnancy BMI, Parity, Gestational Age at Visit; β Estimates and 95% Confidence Intervals Corresponding to Percentage Change in Bioactive Lipids associated with a Doubling in PFAS for PROTECT within-cohort models, unadjusted; β Estimates and 95% Confidence Intervals Corresponding to Percentage Change in Bioactive Lipids associated with a Doubling in PFAS for PROTECT within-cohort models, adjusted for Maternal Age, Maternal Education, Pre-Pregnancy BMI, Parity, Gestational Age at Visit; β Estimates Corresponding to Percentage Change in Bioactive Lipids associated with a 1-Quartile increase in log-transformed PFAS relative to first quartile for random intercept model across all 3 cohorts, adjusted for Maternal Age, Maternal Education, Pre-Pregnancy BMI, Parity, Gestational Age at Visit; β Estimates Corresponding to Percentage Change in Bioactive Lipids associated with a 1-Quartile increase in log-transformed PFAS relative to first quartile for random intercept model across CiOB & IKIDS, adjusted for Maternal Age, Maternal Education, Pre-Pregnancy BMI, Parity, Gestational Age at Visit; Quantile g-computation β Estimates Corresponding to Percentage Change in Bioactive Lipids as a Result of Simultaneous 1-Quartile Increase in All Log-Transformed PFAS, Model adjusted for Maternal Age, Maternal Education, Pre-Pregnancy BMI, Parity, Gestational Age at Visit, and Cohort; Comparison of Mixture Effects between Main Quantile G-Computation Results and Sensitivity Analysis Performing Stepwise Inclusion of Covariates; Comparison of Mixture Effects between Main Quantile G-Computation Results and Sensitivity Analysis Performing Multiple Imputation Supplementary data to this article can be found online (Appendix B. Supplementary Tables S1–S22) (XLSX)
ECHO OIF PFAS Lipids R Script (TXT)

## Notes

**Role of the Funder**: The sponsor, NIH, participated in the overall design and implementation of the ECHO Program, which was funded as a cooperative agreement between NIH and grant awardees. The sponsor approved the Steering Committee-developed ECHO protocol and its amendments including COVID-19 measures. The sponsor had no access to the central database, which was housed at the ECHO Data Analysis Center. Data management and site monitoring were performed by the ECHO Data Analysis Center and Coordinating Center. All analyses for scientific publication were performed by the study statistician, independently of the sponsor. The lead author wrote all drafts of the manuscript and made revisions based on coauthors and the ECHO Publication Committee (a subcommittee of the ECHO Steering Committee) feedback without input from the sponsor. The study sponsor did not review nor approve the manuscript for submission to the journal.
