# Supplementary material for: Cross-Sectional Associations between Prenatal Per- and Poly-Fluoroalkyl Substances and Bioactive Lipids in Three Environmental Influences on Child Health Outcomes (ECHO) Cohorts
Source: Environ Sci Technol. 2024 May 1;58(19):8264–77. doi: 10.1021/acs.est.4c00094 (PMC11097396; doi:10.1021/acs.est.4c00094)
Supplement: Supplementary file 1 — es4c00094_si_001.pdf [file es4c00094_si_001.pdf]

Supplementary Figures for

Cross-sectional Associations Between Prenatal Per- And Poly-Fluoroalkyl Substances and Bioactive Lipids in Three Environmental influences on Child Health Outcomes (ECHO) Cohorts

Authors: Himal Suthar, Tomás Manea, Dominic Pak, Megan Woodbury, Stephanie M. Eick, Amber Cathey, Deborah J. Watkins, Rita S. Strakovsky, Brad A. Ryva, Subramaniam Pennathur, Lixia Zeng, David Weller, June-Soo Park, Sabrina Smith, Erin DeMicco, Amy Padula, Rebecca C. Fry, Bhramar Mukherjee, Andrea Aguiar, Sarah Dee Geiger, Shukhan Ng, Gredia Huerta-Montanez, Carmen Vélez-Vega, Zaira Rosario, Jose F. Cordero, Emily Zimmerman, Tracey J. Woodruff, Rachel Morello-Frosch, Susan L. Schantz, John D. Meeker, Akram Alshawabkeh, Max T. Aung and on behalf of Program Collaborators for Environmental Influences on Child Health Outcomes*

Corresponding author:
Max T. Aung
University of Southern California, Keck School of Medicine
Department of Population and Public Health Sciences
SSB 225R
1845 N Soto St., Los Angeles, CA, 90032
Email: maxaung@usc.edu

This file includes Supplemental Figures S1, S2, and S3A-S3B, totaling 5 pages

Table of Contents:

Supplemental Figure 1. Flow diagram of final sample selection across CiOB, IKIDS, and PROTECT cohorts
Supplemental Figure 2. Directed acyclic graph of the relationships between maternal per- and poly-fluoroalkyl substances and bioactive lipids
Supplemental Figure 3. Correlation matrix of bioactive lipids and high-detect PFAS in the study cohorts.
- **A.** CiOB Cohort
- **B.** IKIDS Cohort
- **C.** ECHO-PROTECT Cohort

Supplemental Figure 1. Flow diagram of final sample selection across CiOB, IKIDS, and PROTECT cohorts.

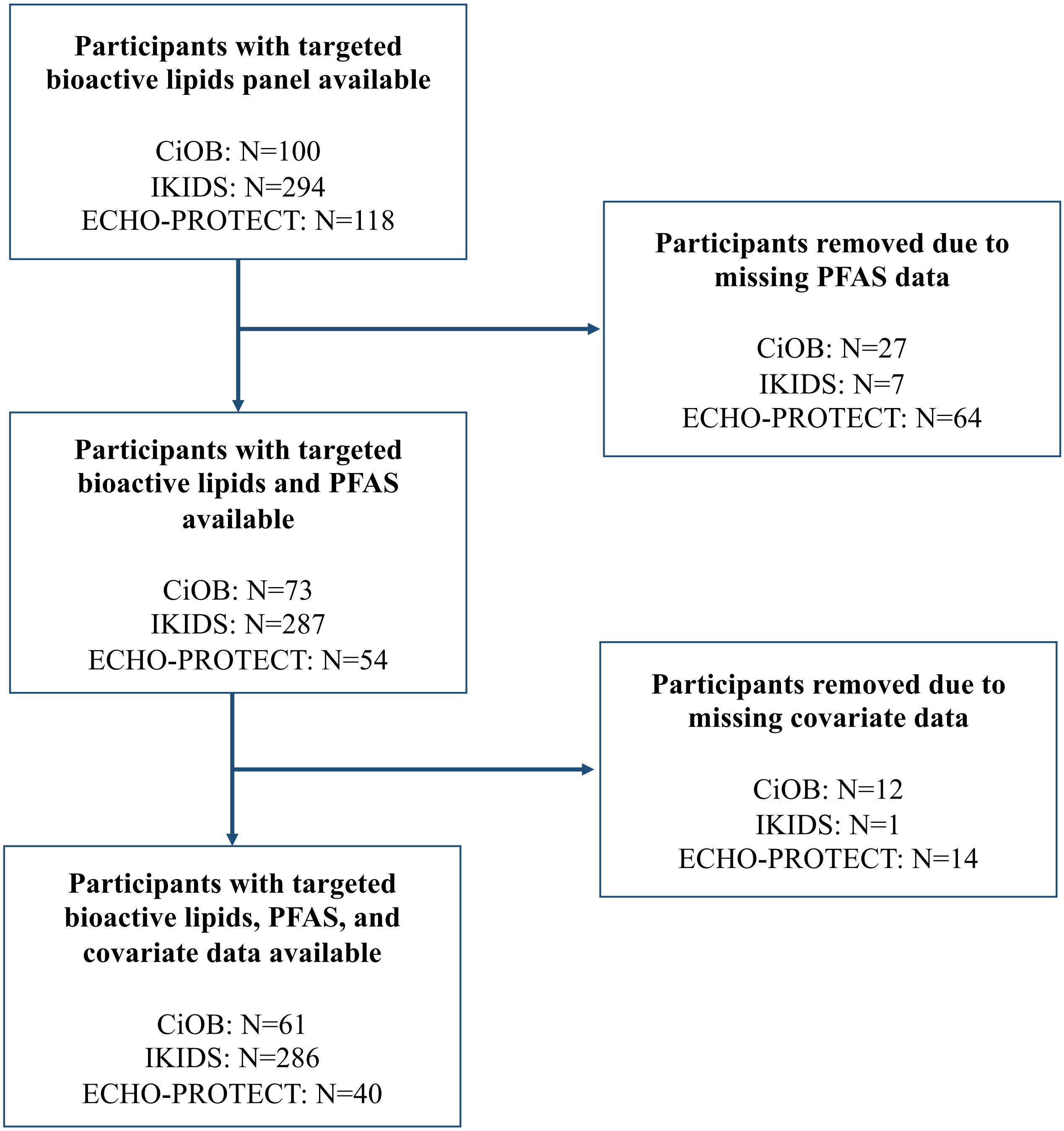

Supplemental Figure 2. Directed acyclic graph of the relationships between maternal per- and poly-fluoroalkyl substances and bioactive lipids.

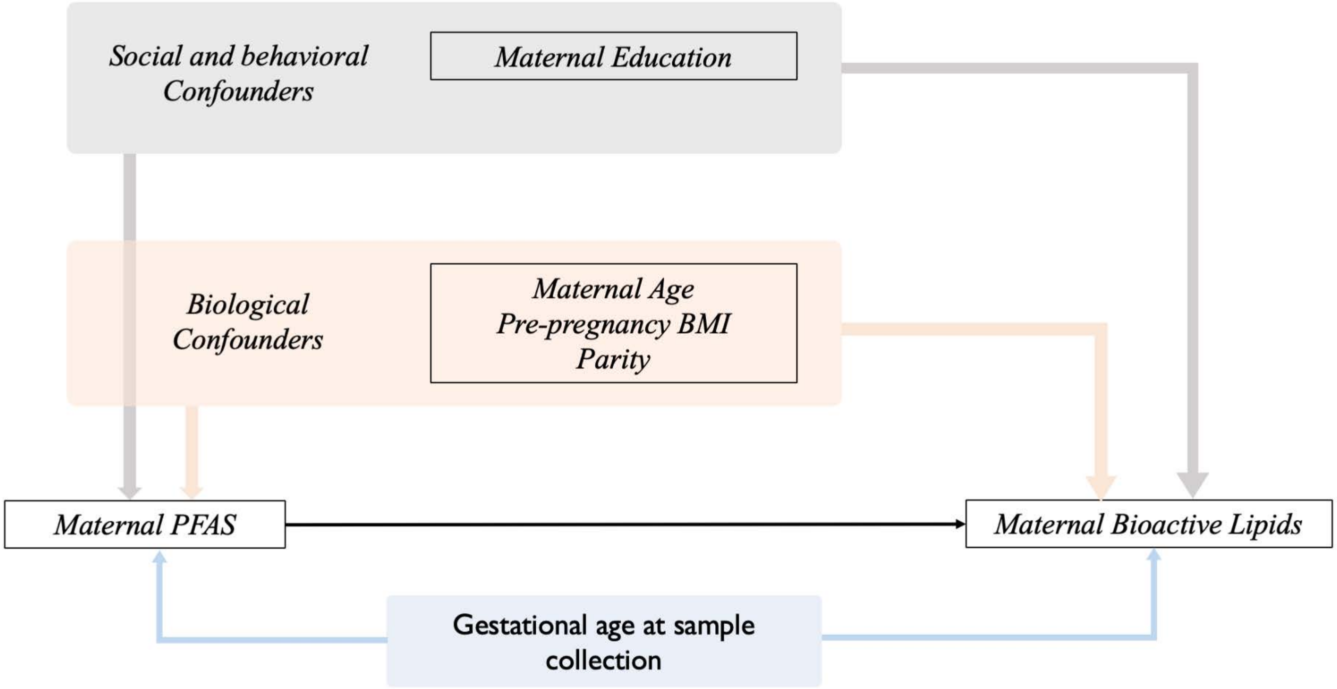

Supplemental Figure 3. Correlation matrix of bioactive lipids and high-detect PFAS in the study cohorts. **(A)** CiOB cohort **(B)** IKIDS cohort **(C)** ECHO-PROTECT cohort

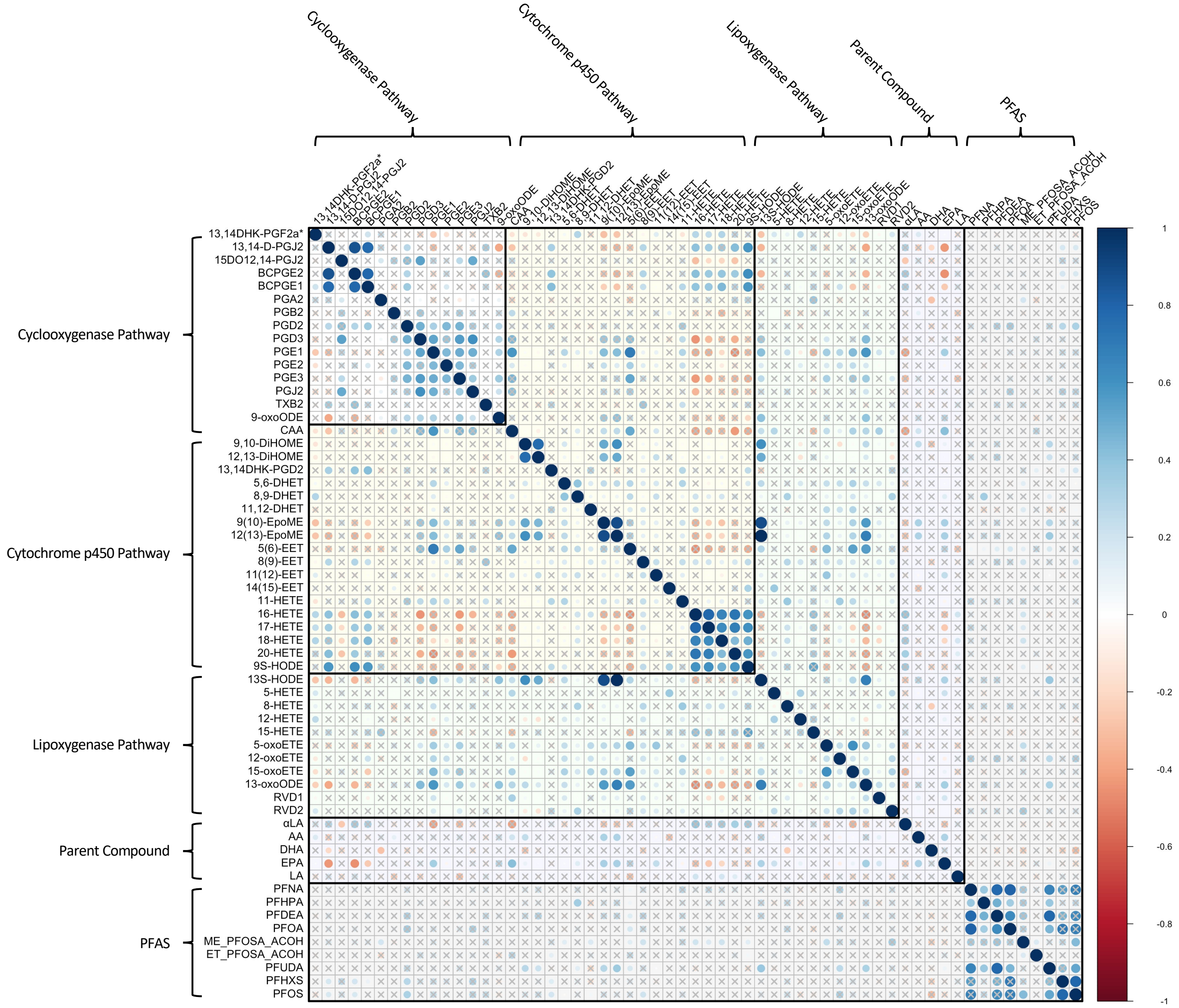

A. Correlation matrix of bioactive lipids and high-detect PFAS in CiOB cohort

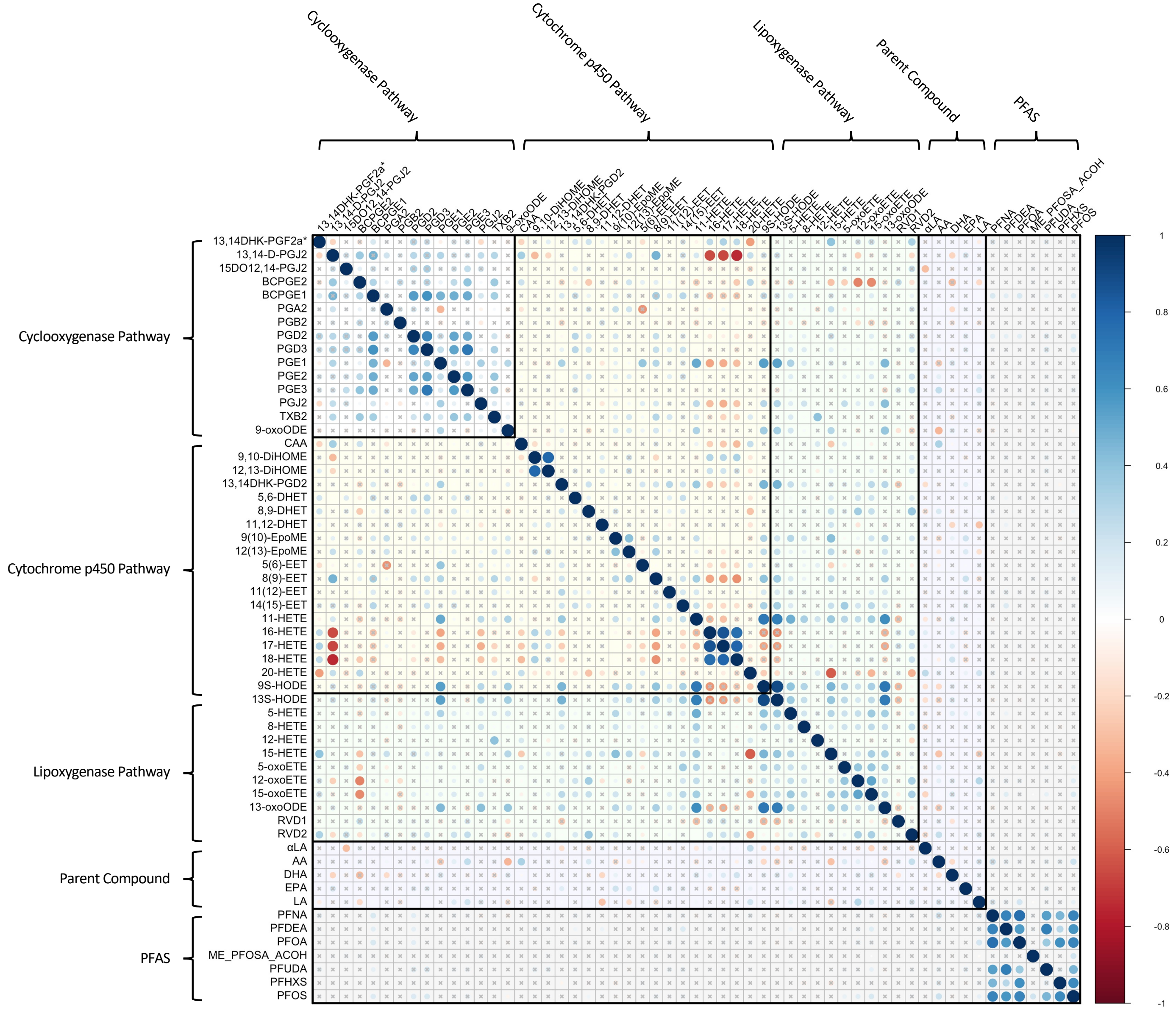

B. Correlation matrix of bioactive lipids and high-detect PFAS in IKIDS cohort

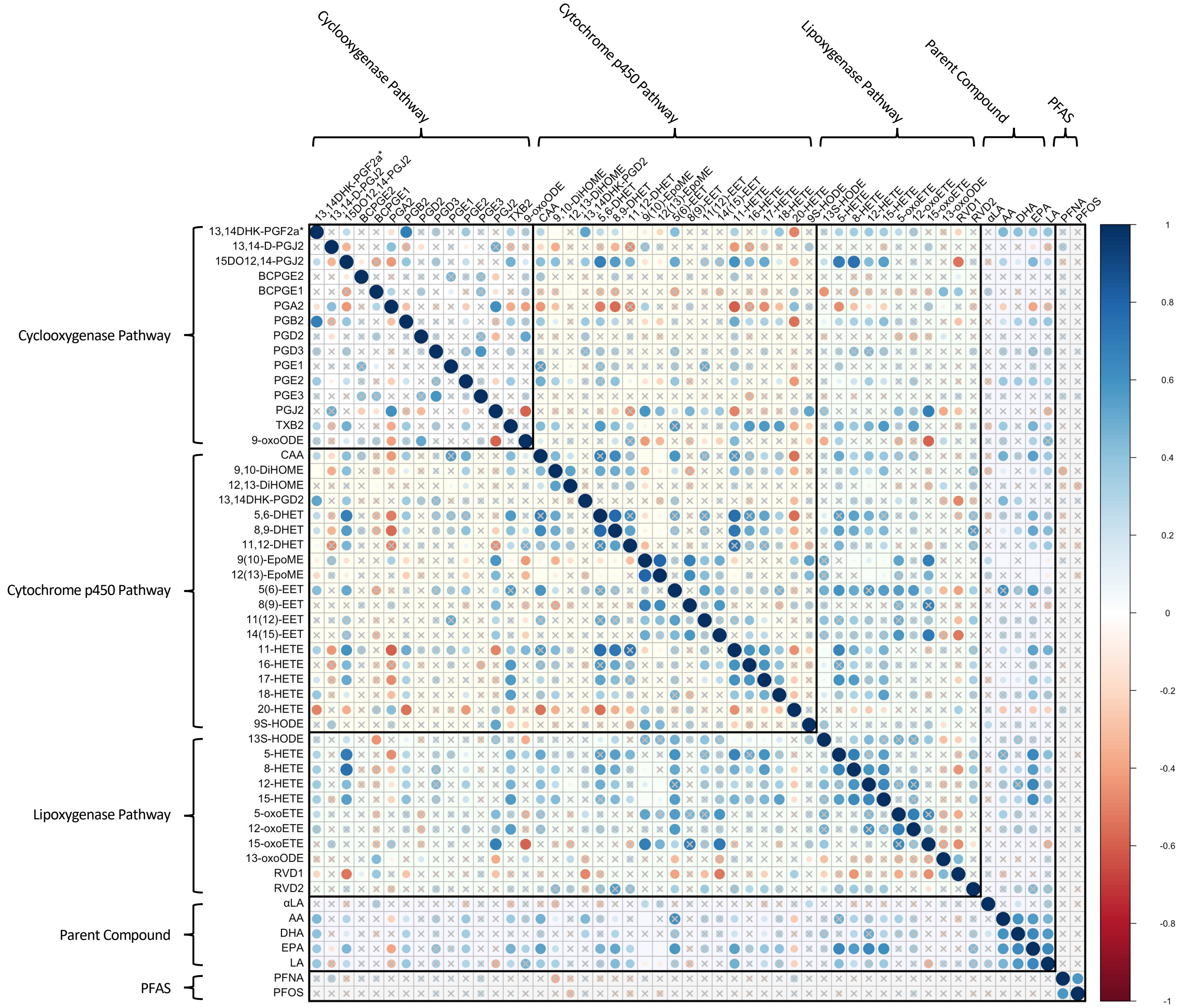

C. Correlation matrix of bioactive lipids and high-detect PFAS in ECHO-PROTECT cohort
